# Methicillin-Resistant *Staphylococcus aureus* (MRSA) Contamination in Bedside Surfaces of a Hospital Ward and the Potential Effectiveness of Enhanced Disinfection with an Antimicrobial Polymer Surfactant

**DOI:** 10.3390/ijerph120303026

**Published:** 2015-03-11

**Authors:** John W. M. Yuen, Terence W. K. Chung, Alice Y. Loke

**Affiliations:** 1 School of Nursing, The Hong Kong Polytechnic University, Yuk Choi Road, Hung Hom, Kowloon, Hong Kong, China; E-Mail: alice.yuen.loke@polyu.edu.hk; 2 Queen Mary Hospital, 102 Pok Fu Lam Road, Hong Kong, China; E-Mail: terencechungwk@gmail.com

**Keywords:** MRSA, staphylococcal infections, surface contamination, environmental contamination, quaternary ammonium chloride, JUC, antimicrobial surfactant

## Abstract

The aim in this study was to assess the effectiveness of a quaternary ammonium chloride (QAC) surfactant in reducing surface staphylococcal contamination in a routinely operating medical ward occupied by patients who had tested positive for methicillin-resistant *Staphylococcus aureus* (MRSA). The QAC being tested is an antibacterial film that is sprayed onto a surface and can remain active for up to 8 h. A field experimental study was designed with the QAC plus daily hypochlorite cleaning as the experimental group and hypochlorite cleaning alone as the control group. The method of swabbing on moistened surfaces was used for sampling. It was found that 83% and 77% of the bedside surfaces of MRSA-positive and MRSA-negative patients respectively were contaminated with staphylococci at 08:00 hours, and that the staphylococcal concentrations increased by 80% at 1200 h over a 4-hour period with routine ward and clinical activities. Irrespective of the MRSA status of the patients, high-touch surfaces around the bed-units within the studied medical ward were heavily contaminated (ranged 1 to 276 cfu/cm^2^ amongst the sites with positive culture) with staphylococcal bacteria including MRSA, despite the implementation of daily hypochlorite wiping. However, the contamination rate dropped significantly from 78% to 11% after the application of the QAC polymer. In the experimental group, the mean staphylococcal concentration of bedside surfaces was significantly (*p* < 0.0001) reduced from 4.4 ± 8.7 cfu/cm^2^ at 08:00 hours to 0.07 ± 0.26 cfu/cm^2^ at 12:00 hours by the QAC polymer. The results of this study support the view that, in addition to hypochlorite wiping, the tested QAC surfactant is a potential environmental decontamination strategy for preventing the transmission of clinically important pathogens in medical wards.

## 1. Introduction

Microorganisms on hospital surfaces can be transmitted to the hands of healthcare workers, patients, and visitors, resulting in cross-infections and epidemics. Despite the implementation of routine cleaning and precautionary measures in most hospitals, effective environmental decontamination methods are still in demand. In recent decades, numerous polymeric surfactant products have been shown to have excellent antimicrobial properties against surface contamination, but none have been tested on hospital surfaces [1,2]. JUC spray is a nano-scale technology formulated with cationic organosilicon quaternary ammonium chloride (OrganoSiQAC) as a major ingredient that is currently being marketed as an FDA-approved invisible hydrogel antimicrobial dressing for wound care. According to a local case report, the JUC spray has also been demonstrated to be effective in managing MRSA-associated skin abscesses [3]. Two recent trials [4,5] have demonstrated a reduction in bacterial burdens from using JUC polymer on critical medical surfaces. These included urinary catheters, where the associated incidence of infection was significantly reduced [5]. The manufacturer of JUC claims that this antimicrobial film stays on animate and non-animate surfaces for up to eight hours [1]. Our research team was therefore particularly interested in investigating the long-acting surfactant capacity of JUC polymer on hospital surfaces and its potential application as an effective decontamination aid. 

In hospitals, surfaces with which patients have close contact or that are highly accessible to patients are more likely to become contaminated. Environmental MRSA contamination has been extensively reported in different areas of a hospital, including in intensive care units [6,7,8,9], burn units [10], isolation rooms [11,12], and general wards [13]. In acute hospital wards, MRSA can be recovered from 1%–27% of surfaces in MRSA-positive patient rooms [14]. However, the incidence of MRSA contamination varies among different hospital ward surfaces, as contamination is influenced by various factors such as the condition of the patient, the ward setting, crowding, and even the sampling method [15,16]. It has been well documented that high-touch surfaces are major reservoirs for MRSA in hospital environments. Of all hospital surfaces, bedside rails in wards occupied by MRSA patients have been identified as the site most frequently contaminated with MRSA [14]. Other frequently contaminated surfaces include bed cranks, overbed tables, bed linens, bedside lockers, bedside trays, pressure cuffs, intravenous pumps, curtains, door handles, keyboards, and floors [6,9,11,13].

The prevalence of hospital-associated MRSA (HA-MRSA) infection varies geographically. Hong Kong is one of the high-prevalence areas in Asia [17]. According to the Asian Network for Surveillance of Resistant Pathogens (ANSORP) study, 57% of all inpatient isolates of *S. aureus* from Hong Kong hospitals were shown to be methicillin resistant [18]. In Hong Kong, healthcare services are provided by the government-supported Hospital Authority. In public hospitals, general wards are typically arranged in the setting of six beds per cubicle. Unlike in many other countries, known cases of MRSA colonization or infection at admission would not be isolated or assigned to single rooms. Rather, such patients would occupy beds at the far end of a ward. Since the surfaces of bedside environments are not considered critical surfaces in terms of contact with mucosal membranes, such surfaces are cleaned with hypochlorite wipes once a day in accordance with the environmental infection control strategies of the U.S. Centers for Disease Control and Prevention (CDC) [15]. Protective barrier precautions, such as the use of gloves and masks, are taken when handling MRSA patients. In view of the high MRSA infection rate in Hong Kong and the approach to dealing with MRSA-positive patients in local medical wards, the primary aim of this study was to evaluate the decontamination effectiveness of JUC polymer on bedside surfaces in a routine-operating medical ward in addition to the daily cleaning hypochlorite wipe and protective barrier precautions. To the best of our knowledge, there has been no epidemiological study thus far on surface MRSA-related contamination in the general wards of Hong Kong hospitals. The degree to which highly accessible bedside surfaces within a medical ward are contaminated with MRSA and other staphylococcal bacteria was assessed before the JUC polymer was evaluated, particularly when the ward was occupied by an MRSA-positive patient. 

## 2. Experimental Section 

A field experimental study was designed to assess the degree of staphylococcal contamination on the bedside surfaces of a medical ward, and the effects of JUC polymer on reducing such contamination. Routine operations continued to be carried out in the ward to reflect the most natural environment for assessment. 

### 2.1. Setting of the Study Hospital Ward

**Figure 1:**
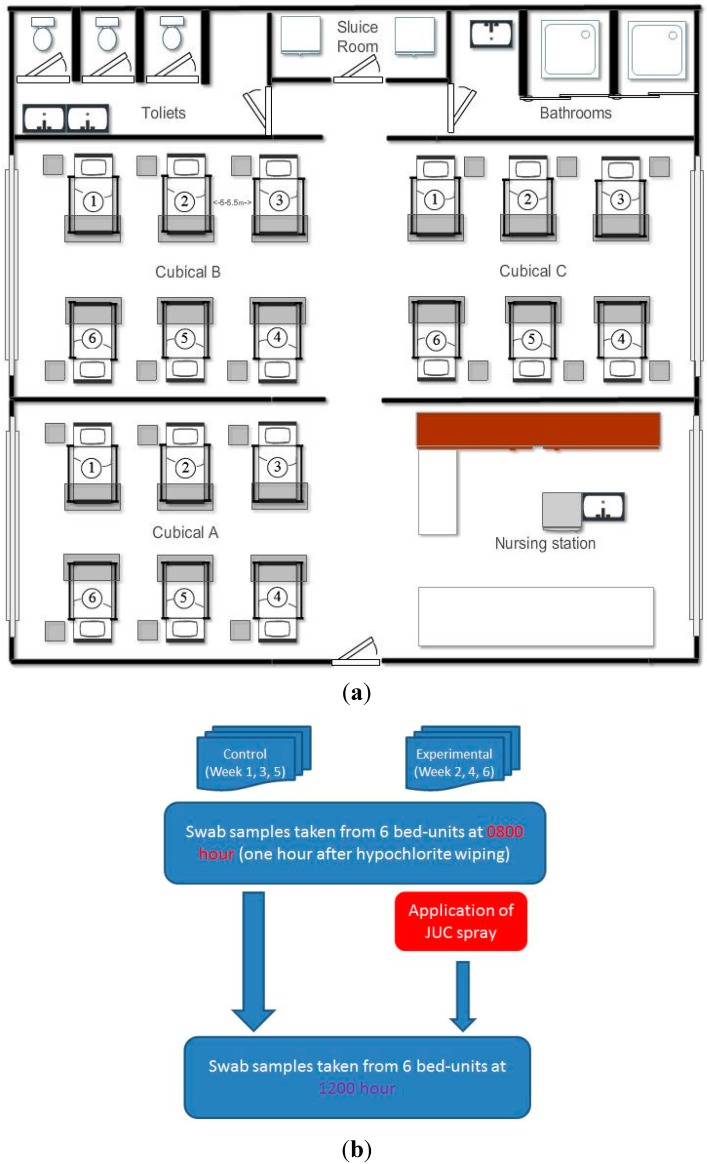
(**a**) Ward layout with three cubicles, and each cubicle consisting of six bed-units. Experiments and environmental sampling were conducted in Cubicle A, as it is separated from the other two cubicles and relatively remote from the sluice room, bathrooms, and toilets. On all sampling days the MRSA patients occupied Bed-unit 1 at the far end. (**b**) Experimental schedule for the study and control arms and to illustrate 6 weeks were required for the whole experiment because there was a week washing out period after each sampling day, in order to avoid the carry-over effects (if any) of the JUC spray.

The study hospital was a large teaching hospital in Hong Kong with a capacity over 1500 beds. The study ward was a male medical ward, consisting of a total of three cubicles each containing six beds, which were fully occupied on all of the days during the study period. The ward and cubicle setting is illustrated in Figure 1a. Cubicle A was designated for this study. Each cubicle was 53 by 60 square meters in area, and each bed-unit occupied 21 by 9 square meters with a bed-to-bed distance of 5 to 5.5 m. An indoor temperature of 24.5 to 25.5 degrees Celsius and humidity of 50 to 55 percent were maintained in the ward. The ward had 24-hour air conditioning with a proper ventilation system, and all of the windows were closed. Sampling to assess the contamination and experiments to evaluate the JUC polymer were carried out only on the days when the cubicle was occupied by one known patient with MRSA infection or colonization for not less than 24 h. In accordance with the usual practice, the MRSA-positive patients occupied bed number one of the cubicle (Figure 1a).

### 2.2. Assessing Staphylococcal Contamination on Bedside Surfaces

Four high-touch bedside surface sites (including the bedside table, the left-side handrail, the right-side handrail, and the overbed rolling table) were selected for the swab sampling. All of the selected sites had been identified as high-touch surfaces using a quantitative approach [19]. The sampling method was tested before the main study was commenced. The pilot results also indicated a remarkable degree of contamination (83% of the cultured surface specimens was found to be positive for staphylococci ranged in average 1 to 27 CFU/cm^2^ per sampling site) at all of the selected sites. Using flocked or macrofoam swabs were found to be a reliable and efficient strategy for recovering bacteria from environmental surfaces [20]. In this study, the “swabbing on moistened surfaces” method was adopted using Puritan^®^ sterile macrofoam tip environmental swab applicators for swab sampling. Swabbing was performed at three random spots of each sampling surface standardized to 10 square centimeters. Hence, a total of 12 swab specimens (three samples per site × four spots) were collected from each bed-unit. The swabbed specimens were inoculated into vials containing 1 mL of sterile water and transferred to Petrifilm^TM^ Staph Express Count Plates (3M, St. Paul, MN, USA).

The degree of contamination in the bed-units occupied by MRSA-positive and MRSA-negative patients was compared. Swab specimens were collected from all bed-units within the cubicle at two time points at 08:00 hours and 12:00 hours, and this was repeated on six separate days over a 6-week period. The time of eight o’clock in the morning was chosen because that is not long after hypochlorite wiping has been done, at 07:00 hours, and most patients are awake following the minimal activities during the night shift within the ward. The hypochlorite solution was freshly prepared according to hospital guidelines by a duty healthcare worker, who also performed the wiping of the whole cubicle under the supervision of a duty nurse to ensure that all surfaces were cleaned. Hence, the 08:00 hours specimens were used as the baseline for comparisons between the MRSA-positive and MRSA-negative surfaces. Furthermore, the 12:00 hours specimens were used to assess natural changes in contamination over the four-hour interval during which routine clinical activities were carried out without disturbance. During the four-hour period before the lunch hours, visitors are generally not allowed to enter the ward, which minimizes disturbances other than those from patients and healthcare workers. However, healthcare professionals outside the ward cannot be prevented from entering the ward. Over the course of the 6-week study, a total of 864 swab specimens (three samples per site × four spots × six beds × two time points × six days) were collected from the designated site surfaces.

### 2.3. Experimental Design for Evaluating the Decontamination Effects of JUC Polymer

In this experiment, the decontamination property of the JUC polymer was evaluated on the same designated sites of the bed-units used for the staphylococcal assessment in the same ward cubicle. The experimental arm tested the JUC spray plus the routine daily cleaning (*i.e.*, sanitization with bleaching water at 07:00 hours), while the control arm tested the routine daily cleaning alone. Regardless of the study arm, swab specimens were collected at 08:00 hours as the baseline and at 12:00 hours for assessing the change in the staphylococcal burden. The JUC dressing spray was gifted by the NMS Technologies Company (Nanjing, China). On the experimental days, the JUC spray was applied to the site surfaces immediately after the baseline specimens had been collected (see Figure 1b). The following spraying method recommended by the manufacturer was followed: the target surface was evenly sprayed at a distance of 10 cm for 5 s to cover the spot area. No dangerous aerosol should be generated when spraying, since the JUC spray is 98% composed of water. The JUC spray was applied to all bed-units within the cubicle and the process was repeated three times over a 3-week period, for a total of 72 spots, *i.e.*, four sites per bed × six beds per cubicle × three experimental days. Since three random swabs were collected from each surface site, there were a total of 216 specimens per experiment or control arm. The swabbed specimens were inoculated into vials containing 1 mL of sterile water and transferred to Petrifilm^TM^ Staph Express Count Plates (3M). To avoid a carry-over effect from the JUC spray, each experimental day was arranged to be at least 7 days apart from the next experimental day. The control arm was also conducted on three separate days. On each experimental and control day, one MRSA-positive patient occupied the bed-unit at the far end, *i.e.*, Bed-unit A1 in Figure 1. The ward staff and healthcare workers were blinded to the experiment in order to ensure that the routine ward activities were not influenced and disturbed. The demographic characteristics of the patients occupying the bed-units, including their MRSA status, are summarized in Table 1.

**Table 1:** Demographic characteristics of patients who occupied the bed-units in the experimental and control days.

Parameters			Experimental Group (n = 18)	Control Group (n = 18)	Paired *t*-Test
Mean Age ± SD (Range)			81.13 ± 7.17 (67–91)	73.44 ± 16.26 (38–92)	0.1041
Total number of patients			18	18	N.A.
Admitted from elderly homes (Number of patients)	Yes		11 (61%)	10 (63%)	N.A.
	No		7 (39%)	6 (37%)	N.A.
Positive culture for MRSA^#^	Wound swab		1	1	N.A.
(Number of patients)	Nasal swab		0	1	
	Sputum		2	1	N.A.
	None		15	15	N.A.
Mean hospitalization days ± SD (Range)			7.81 ± 4.35 (2–16)	6.75 ± 2.95 (1–14)	0.4395

Note: A total of 18 patients (with three MRSA-positive patients in three separate days) were involved in each arm of the study (experiment: JUC spray + standard cleaning verses control: standard cleaning). They were studied over three separate days (each 7 days apart). On each day, all six beds were fully occupied, with one MRSA carrier and five non-MRSA carriers within the ward cubicle.

### 2.4. Bacterial Culture and Identification

Within one hour following the collecting of samples, all inoculated Staph Express Count Plates were sent to the microbiology laboratory of the study hospital for incubation and further analysis. All of the plates were incubated at 37 degrees Celsius for 24–48 h. Red-violet colonies were counted as positive for staphylococcal growth and expressed in Colony-forming Units per Centimeter Square (cfu/cm^2^). Since each swab sample was collected from a surface area of 10 cm^2^, the CFU/Petrifilm actually represented the growth of staphylococcal colonies from each swab specimen covering 10 cm^2^ surface area. The minimum detection (countable) limit of the sampling technique was defined as 1 cfu/cm^2^. Positive growth on the plates was followed up using the coagulase test for differentiating coagulase-positive and coagulase-negative staphylococci (CNS). Subculture of the coagulase-positive staphylococci (red-violet colonies were randomly picked from Staph Express Count Plates) was performed in Mueller-Hinton agar supplemented with 4% w/v sodium chloride, and their sensitivity to oxacillin was assessed by using the disc diffusion test as recommended by the U.S. Centers for Disease Control and Prevention (http://www.cdc.gov/HAI/settings/lab/lab_mrsa.html). MRSA strains are resistant to all β-lactam antibiotics, but typically oxacillin resistant. Therefore, in this study, methicillin-resistant *Staphylococcus aureus* (MRSA) strains were identified and differentiated from the methicillin-sensitive *Staphylococcus aureus* (MSSA) strains. According to the technical bulletin and user instruction of the JUC spray dressing, the QAC solution will be solidified immediately after contacting the air after spraying onto any surface, and its bactericidial property is exerted by the physical electrostatic force generated between the positively-charged coating surface and the negatively-charged cell wall or membrane of the organisms. The coating adhered on the surface will then be water-proof and stay for up to 8 h. Such coating should not be removed from the sampling surface when performing the swab. Furthermore, physical bactericidal activity will be lost even if the coating is being released in solution, and therefore, neutralizer is not required for the specimen collection procedure from the bedside surface with the JUC coating and residual QAC is not a concern that could affect the microbiological results.

### 2.5. Ethical Considerations

The experimental procedure was reviewed and approved by the ethics committee of the Hong Kong Polytechnic University as well as that of the study hospital. This study involved only the environmental surfaces around the bed-units inside a ward cubicle, where neither patients nor ward staff were involved. The JUC spray is an FDA-approved wound care product that causes no harm to humans. At admission, the MRSA status of the patients was confirmed according to the patients’ history and to information provided by the hospital infections control team, and this information was not disclosed anywhere to anyone.

## 3. Results

**Figure 2:**
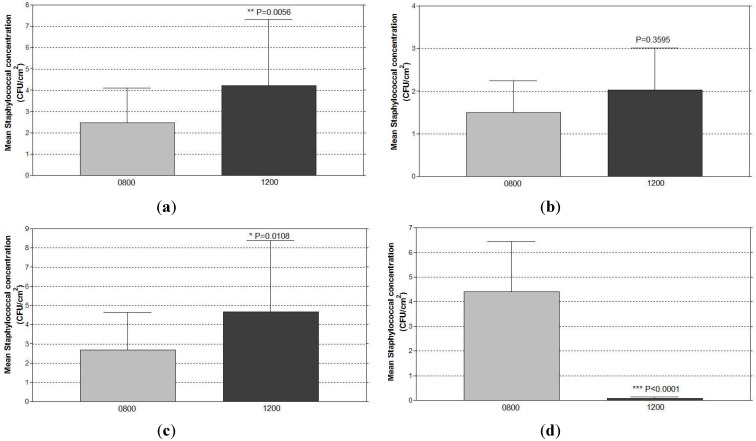

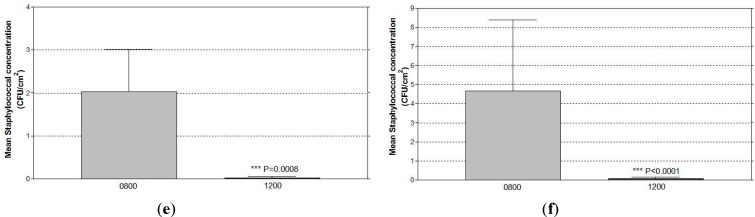
The increase in staphylococcal concentrations recovered from the bedside surfaces of (**a**) all control (received hypochlorite cleaning only) bed-units irrespective of the patients’ MRSA status (n = 36). (**b**) MRSA-positive patients (n = 6). (**c**) MRSA-negative patients (n = 30). The reduction in staphylococcal concentrations recovered from the bedside surfaces 4 hours after the application of JUC spray of (**d**) all bed-units irrespective of the patients’ MRSA status (n = 18). (**e**) MRSA-positive patients (n = 3). (**f**) MRSA-negative patients (n = 15).

At 08:00 hours, positive staphylococcal growth was recovered from 83% (five out of six) and 77% (23 out of 30) of the bedside surfaces occupied by MRSA-positive and MRSA-negative patients, respectively in the studied ward cubicle (Table 2). Irrespective of MRSA carrier status, both coagulase-positive (CPS) and coagulase-negative staphylococci (CNS) were recovered from all bed-units. Three random swab specimens were collected at 08:00 hours (an hour after hypochlorite cleaning) from each bedside surface of each bed-unit, thus a total of 108 swabs from each bedside surface site tested. Majority of the sampled bedside surfaces were negative for the bacterial growth (4.6%–11.1% positive for CNS; 2.8%–9.3% positive for MSSA; and 7.4%–17.6% positive for MRSA as summarized in Table 3). Among those surfaces with positive growth, the colony numbers recovered for CNS, MSSA, and MRSA were ranged 1–16, 1–193, and 1–276 cfu/cm^2^, respectively. Irrespective of the swabbing site, CNS, MSSA, and MRSA were recovered from 44%, 28%, and 56% of all bed-units sampled. The mean CNS concentrations in the bed-units of the MRSA-positive and MRSA-negative patients were 1.9 cfu/cm^2^ and 1.6 cfu/cm^2^, respectively. From the bed-units of the MRSA-positive patients, the ratio of CPS and CNS was found to be 2:4, and all CPS isolates were revealed to be oxacillin resistant with an overall burden of 3.9 cfu/cm^2^. On the other hand, from the MRSA-negative bed-units, the CPS isolates were a mixture of MSSA and MRSA. Half of the CPS isolates were revealed to be oxacillin resistant with a mean concentration of 7.9 cfu/cm^2^, which was significantly (*p* < 0.05) heavier than the amount recovered from the MRSA-positive bed-units. Overall, the staphylococcal distribution among CNS, MSSA, and MRSA was in the ratio of 2:6:8. Reference to the layout of the testing ward cubicle (Figure 1a), heavier growths were found to be at beds next to the MRSA-positive bed (*i.e.*, beds 2 and 6). Bed 4 was also observed to have relatively heavy growth. However, none of the beds had growth that was statistically different from that of other beds (data is not shown). Among all control bed-units received only hypochlorite cleaning, the mean staphylococcal contamination increased significantly (*p* < 0.01) by 80% from 08:00 to 12:00 hours (Figure 2a). 

**Table 2:** A comparison of the concentrations and types of staphylococcal bacteria recovered from the bedside surfaces of bed-units occupied by MRSA-positive and MRSA-negative patients at 08:00 hours.

Types of Staphylococci and Sampling Sites		Mean cfu/cm^2^ ± Standard Error of Mean (SEM)			
		MRSA-Positive		MRSA-Negative	*p* Value
		Bed-Units (n = 6)		Bed-Units (n = 30)	
CNS					0.1953
Bedside table		N.D.		0.04 ± 0.03	
Left-side handrail		0.39 ± 0.33		0.12 ± 0.09	
Right-side handrail		1.94 ± 1.13		1.20 ± 1.13	
Overbed rolling table		N.D.		0.29 ± 0.25	
					
MSSA					Undetermined
Bedside table		N.D.		0.48 ± 0.39	
Left-side handrail		N.D.		1.94 ± 1.83	
Right-side handrail		N.D.		3.08 ± 2.17	
Overbed rolling table		N.D.		0.02 ± 0.01	
					
MRSA					0.0392
Bedside table		N.D.		0.68 ± 0.63	
Left-side handrail		0.72 ± 0.72		6.37 ± 4.03	
Right-side handrail		1.11 ± 0.98		0.57 ± 0.30	
Overbed rolling table		2.11 ± 1.41		0.28 ± 0.14	

Notes: A total of 36 bed-units were sampled in 6 separate days at 08:00 hours. The studied cubicle was fully occupied in all of those days, and only one MRSA-positive patient was included in each sampling day. The statistical difference in MSSA loadings between the MRSA-positive and MRSA-negative could not be determined, because the count for one comparison group was zero. N.D. = Non-detectable which means colony is absent in the petrifilm plate. The detection limit of the sampling technique was 1 cfu/Petrifilm (which is equivalent to cfu/cm^2^). Some of the mean cfu/cm^2^ values presented in this table were smaller than the detection limit as influenced by a large number of 0 (non-detectable) values (refer to Table 3 for the percentage of the positive culture at each sampling site).

**Table 3:** Number, percentage and the range of colony numbers of the three staphylococcal species recovered from the surface swabbing sites at 08:00 hours (an hour after the hypochlorite cleaning).

Types of Staphylococci and Parameters		Site for Surface Swabbing (n = 108 for Each Site)			
		Bedside Table	Left-Side Handrail	Right-Side Handrail	Overbed Rolling Table
CNS					
	Number (%) of positive culture	5 (4.6)	6 (5.6)	12 (11.1)	7 (6.5)
	Range of cfu/cm^2^	1–2	1–9	1–16	1–14
	among positive culture				
					
MSSA					
	Number (%) of positive culture	7 (6.5)	10 (9.3)	4 (3.7)	3 (2.8)
	Range of cfu/cm^2^	1–36	1–164	1–193	1–2
					
MRSA	among positive culture				
	Number (%) of positive culture	8 (7.4)	19 (17.6)	19 (17.6)	12 (11.1)
	Range of cfu/cm^2^	1–23	1–276	1–75	1–26
	among positive culture				

The same trend was observed in the bed-units of MRSA-positive and negative patients; however, the increase in staphylococcal contamination in the MRSA-negative beds (from 2.8 to 4.8 cfu/cm^2^) was approximately four times that in the MRSA-positive bed-units (from 1.5 to 2.0 cfu/cm^2^) (Figure 2b,c). The staphylococcal strain that increased after the four-hour period was mainly coagulase positive and sensitive to oxacillin, and hence was MSSA.

With the focus on oxacillin resistance, sites of contamination were shown to be different among the bed-units of MRSA-positive and negative patients. The surface of the left-side handrail was found to be the most heavily contaminated site, containing 84% of all MRSA isolates recovered from the MRSA-negative bed-units. Indeed, the left-side handrail was found to have an MRSA concentration 11-fold significantly (*p* < 0.05) higher than that of the right-side handrail. However, with regard to the MRSA-positive bed-units, 54% of the MRSA isolates were recovered from the overbed rolling table surface as the dominant site of contamination. The mean MRSA concentration on the surface of the right-side handrail was found to be slightly higher (albeit statistically insignificant) than that on the surface of the left-side handrail, while none was recovered from the bedside table specimens. 

As shown in Table 4, over the four-hour interval, the number of bed-units contaminated by staphylococci was dramatically reduced from 78% to 11% following the application of JUC polymer. Only two out of the 18 JUC-applied bed-units (11%) still tested positive for culture and all isolates were identified as CNS, while none of these bed-units had isolated positive cultures of MSSA and MRSA. In the experimental group, the mean staphylococcal concentration of the bedside surfaces had dropped significantly (*p* < 0.0001) from 4.4 ± 8.7 CFU/cm^2^ at 08:00 hours to 0.7 ± 0.26 cfu/cm^2^ at 12:00 hours due to the use of the QAC polymer (Figure 2d). The same trend of reduction was observed irrespective of the MRSA status of the patients (Figure 2e,f).

## 4. Discussion

The novelty of this study lies in the significant reduction in staphylococcal contamination on the bedside surfaces of the medical ward due to the use of the JUC polymer. To the best of our knowledge, this is also the first study to report on the situation of staphylococcal contamination in the general ward environment of hospitals in Hong Kong. It is also the first study to compare staphylococcal contamination between bed-units occupied by MRSA-positive and MRSA-negative patients. 

**Table 4:** Number of bed-units that showed a positive growth in staphylococcal bacteria according to the staphylococcal types recovered from the specimens collected four hours after the application of JUC spray.

Bed-Units with Positive Culture and Types of Staphylococci		No. of Bed-Units (%)	
		Control	Experimental
		(Hypochlorite Cleaning)	(JUC + Hypochlorite Cleaning)
Bed-units of MRSA carrier (n = 3)			
	Positive culture	3 (100%)	0 (0%)
	CNS	1 (33%)	0 (0%)
	MSSA	2 (67%)	0 (0%)
	MRSA	1 (33%)	0 (0%)
			
Bed-units of non-MRSA carrier (n = 15)			
	Positive culture	11 (73%)	2 (13%)
	CNS	2 (13%)	2 (13%)
	MSSA	8 (53%)	0 (0%)
	MRSA	3 (20%)	0 (0%)
			
All bed-units (n = 18)			
	Positive culture	14 (78%)	2 (11%)
	CNS	3 (17%)	2 (11%)
	MSSA	10 (56%)	0 (0%)
	MRSA	4 (22%)	0 (0%)

Note: The experimental arm consisted of the application of JUC spray in addition to daily hypochlorite wiping, while the control arm consisted of daily hypochlorite wiping alone. A total of 18 bed-units were studied for each arm.

### 4.1. Staphylococcal Contamination on the Bedside Surfaces of Bed-Units in the Studied Ward 

Our results indicated that a large number (about 80%) of bedside surfaces was contaminated and that oxacillin resistance was prevalent, occurring not only around the beds of MRSA-positive patients but also of MRSA-negative patients, at one hour (08:00 hours) and five hours (12:00 hours) after a hypochlorite wiping (performed at 07:00 hours). The results reflected the natural contamination within the ward where hypochlorite cleaning was regarded as a routine practice, and authors are aware that it may have affected the staphylococcal identity and proportionate isolation. Surface MRSA contamination was commonly reported in bed-units or rooms occupied by MRSA infected or colonized patients [9,13,14]. With the current study design, with its particular emphasis on staphylococcal contamination in the natural clinical environment and on avoiding disturbances to routine activity and staff duties, there could have been several important sources for the MRSA contamination. First, the MRSA-positive patient could have been a stable source within the cubicle. Creamer and colleagues [21] revealed that patients could shed MRSA, more frequently early in the morning, which could spread in the surrounding air to other hospital surfaces. However, a sophisticated genotyping analysis is needed to confirm that the MRSA isolates were shed from the MRSA-positive patients and dispersed within the ward. The activities of the nursing station might also be a potential source of heterogeneous contamination. However, such activities would also be considered part of the natural ward environment. Although, in normal circumstances visitors were generally not allowed to enter the ward during the four-hour interval, during the operation of the ward visitors could not be completely banned. Therefore, visitors and staff could also be staphylococcal carriers, and staphylococcal bacteria can survive in the environment for months [14]. Hospital staff are known to be the major vehicle of bacterial transmission during routine patient care [22,23]. Ward activities, such as ward rounds, clinical activities (resuscitation, sampling, *etc.*) and bed-making could all contribute to the environmental dispersal of staphylococci. This is consistent with the results of this study, in which the overall surface staphylococcal burden from the sampled sites increased by 80% from 08:00 to 12:00 hours, when routine ward and clinical activities were maintained. Regarding the CNS strains that were recovered from all surfaces, the most clinically important CNS causing nosocomial infections is known to be *Staphylococcal epidermidis*, which is frequently sourced from the skin normal flora of patients and healthcare workers [24]. Nonetheless, the attitudes and beliefs of the cleaning staff are considered as an additional factor that some staff may carried out the cleaning more effectively for the bed-unit of the known MRSA-positive patients. This factor cannot be ignored in the current study. 

Hospital ward environments, especially high-touch surfaces, are rich reservoirs for the transmission of many microorganisms. The current results were consistent with those of previous studies reporting that handrails are the most frequently contaminated surfaces within a ward environment [14]. Handrails and overbed rolling tables were the dominant sites of contamination in the bed-units occupied by the non-MRSA and MRSA patients. The advantages of wiping high-touch surfaces daily with sporicidal agents such as bleaching water have been experimentally demonstrated [25]. Although hypochlorite disinfectants are well known for being able to eliminate a wide spectrum of bacteria, including MRSA, the current results show the drawbacks of relying on hypochlorite wipes to decontaminate hospital environments. A local study reported on the failure of disinfection efforts using hypochlorite wiping by demonstrating the presence of MRSA in bedside rails after hypochlorite wiping. A possible cause was the failure to thoroughly rinse the wipe, as suggested by the presence of bacteria in the wipe before wiping was carried out [26]. However, this study did not set out to assess the effectiveness of hypochlorite wiping. As no sampling was carried out prior to cleaning and the effectiveness of hypochlorite wiping should not be judged in current study. However, results herein suggested that one-off wiping with hypochlorite will not prevent recontamination of the surface over time. Furthermore, it is a fact that hypochlorite agents are easily inactivated by the presence of biofilm formed by bacteria and contamination by organic compounds in the hospital environment [8], and that they only offer immediate but not long-lasting antimicrobial activity, which is a non-modifiable property. Hospital wards may consider increasing the frequency with which they carry out hypochlorite wiping during the day.

### 4.2. Significant Reduction in Surface Staphylococcal Burdens from Using the Antimicrobial Coating

The results clearly indicate that the application of JUC polymer on bedside surfaces effectively reduces both the incidence of staphylococcal contamination and bacterial concentration. The liquid preparation of JUC solidifies immediately when upon contact with the surface of skin or any fabric to form a two-sided film. The bonded film adheres firmly to the surface, and the positively charged film attracts the negatively charged cell walls and membranes of microorganisms to exert electrostatically destructive killing effects [1]. So far, *in vitro* cytotoxicity ranging from 99 to 100 percent has been tested on pathogenic microorganisms including *Staphylococcus aureus*, *Treponena pallidum*, *Pseudomonas aeruginosa*, *Gonococcus*, *Colibacillus*, *Candida albicans*, and SARS coronavirus [27]. The results of this study suggest that the JUC polymer has a long-lasting antimicrobial activity of at least four hours after application. The manufacturer has claimed that its antimicrobial properties last for eight hours, which requires further confirmation. Regarding the toxicity, different tests were performed and JUC polymer has been proven to be safe for use directly on wounds and on the critical surfaces of medical devices [1,27]. Toxicity tests have been performed on mice and rabbits, and the lethal dose 50 (LD_50_) was determined as >10,000 mg/kg, which is essentially non-toxic. In particular it has no irritation to skin and eyes [27]. These findings support the view that the JUC spray should be safe to use for environmental decontamination in hospitals.

Several researchers [28,29] have recommended the use of quaternary ammonium compound (QAC)-based antimicrobial coating on high-touch surfaces. However, a recent study has reported the occurrence of an antiseptic-resistant gene among Hong Kong nurses, which reduces the biocide susceptibility of QAC in staphylococcal organisms [30]. This aspect must be carefully addressed before the JUC spray and other QAC-based surfactants can be further implemented as an effective environmental surface decontamination aid. Despite this, as mentioned above, the antimicrobial activity of JUC spray is exerted by the physical electrostatic force generated between the positively charged coating surface and the negatively charged cell surface, which does not involve any biological or chemical mechanism that may develop the resistance. Nonetheless, the issue of resistance cannot be ignored, and the antimicrobial activity of JUC coating should also be tested against other important hospital-associated organisms such as *Pseudomonas aeruginosa* and multidrug resistant (MDR) gram-negative organisms including *Stenotrophomonas maltophilia* and vancomycin-resistant enterococci in the hospital environment. Such surface treatment should be abandoned if the MDR gram-negatives appear and cause problem for patients. To the best of our knowledge, this is the first study that has been conducted to evaluate the effectiveness of JUC spray as an enhanced surfactant disinfectant in addition to hypochlorite wiping in the general ward environment. The results were obvious on Staphylococci including the coagulase-negative and oxacillin-resistant strains, which warrants a large-scale investigation involving more hospital environments. 

## 5. Conclusions

The application of JUC OrganoSiQAC-based surfactant as a antimicrobial coating was found to be effective in reducing the incidence and bacterial concentrations of bedside staphylococcal contamination. It exerted long-lasting antimicrobial activity for at least four hours after application. The finding supports the application of JUC spray as a potential environmental decontamination strategy to prevent the transmission of clinically important pathogens in medical wards. 
